# Challenges and Issues of Anti-SARS-CoV-2 Vaccines

**DOI:** 10.3389/fmed.2021.664179

**Published:** 2021-05-14

**Authors:** Sophie Blumental, Patrice Debré

**Affiliations:** ^1^Pediatric Infectious Disease Unit, Hôpital Universitaire des Enfants Reine Fabiola, Université Libre de Bruxelles, Bruxelles, Belgium; ^2^Immunology Department, APHP, Sorbonne Université CIMI (Inserm U1135), Hôpital Pitie Salpêtrière, Paris, France

**Keywords:** vaccination, COVID-19, SARS-CoV-2, prevention strategy, vaccine formulation

## Abstract

At the beginning of 2021, anti-SARS-CoV-2 vaccination campaigns had been launched in almost 60 countries with more than 500 million doses having been distributed. In addition to the few vaccines already in use, many other candidates are in preclinical phases or experimental stages in humans. Despite the fact that the availability of anti-SARS-CoV-2 vaccine constitutes a major advance and appear to be the only way to control the pandemic, some investigation remains to be carried out, and this is notably concerning the impact on transmissibility, the duration of the conferred protection in the mid- and long term, the effectiveness against present and future viral mutants, or the ideal schedule that should be applied. In this paper, we review the circumstances that facilitated such a rapid development of anti-SARS-CoV-2 vaccines and summarize the different vaccine platforms under investigation as well as their present results and perspectives in different settings. We also discuss the indications of vaccination under special conditions, such as a history of previous COVID-19 infection or belonging to extreme age categories like children and elderly. Overall, this review highlights the multiple challenges to face if aiming to find a global solution to the pandemic through high vaccination coverage all over the world.

## Introduction

For more than 1 year, SARS-CoV-2 has beenspreading all over the world creating a huge burden of disease with millions of cases of infection and thousands of deaths recorded every day (1).

Even though significant advances have been made in patient management, notably thanks to better understanding and treatment of pulmonary and thrombo-embolic lesions, there is currently no universally approved viral treatment, making until recently from physical distancing and hygiene measures the only means of slowing down the pandemic but at heavy psychosocial, educational, medical and economical costs. While the third wave is ongoing in Europe and an upsurge of cases is observed due to new variants issued from neighboring countries, there is rising hope to control the pandemic thanks to the arrival of the awaited vaccines (2, 3). As of early April 2021, SARS-CoV-2 vaccination campaigns have been launched in roughly 60 countries with more than 500 million doses having been administered globally.

Worldwide, outstanding resources have been deployed to support vaccine development by recruiting thousands of researchers, using high technology, and calling for important financial subsides. Though the availability of vaccines is unanimously considered to be a dramatic progress among scientists, much uncertainty and questions remain inside the general population; these are easily understandable regarding the innovative techniques applied, the uncommon rapidity of commercialization, and the daily flow of conflicting information delivered by the media.

In this setting, we aim to clarify the scientific background that allowed for such a rapid development of a vaccine, to provide a summary of the different formulations available, to discuss the perspectives of vaccination campaigns, and to highlight how challenging such a vaccination program could be in the setting of a pandemic due to a new pathogen.

Our literature review was mainly based on peer-reviewed articles listed on a platform developed by the French Agency for Research on AIDS, Hepatitis, and Emerging Infectious Diseases that selects on a weekly basis the most relevant papers published on COVID-19 vaccines and therapeutics in high-ranked journals of choice. Moreover, we gave much consideration to all scientific information provided by the European Centre of Disease Control and Prevention (ECDC) as well as the World Health Organization (WHO) from which we consulted the website sections dedicated to professionals on weekly basis. Based on these two major sources, preprints papers that were judged to be reliable and highly relevant in context were also included.

## Background and Opportunities

According to the WHO (2), as of April 1, 2021, there were no <84 vaccine candidates in clinical evaluation, 184 candidates in preclinical evaluation, and more than 100 vaccine studies. If so many vaccine candidates are close to the marketing stage only 15 months after the first manifestations of COVID-19, this high-speed development has been facilitated by numerous circumstances and opportunities that are detailed below.

### Background From Previous Studies on Other Coronaviridae

Until recent work against SARS-CoV-2, there was no vaccine approved for human use against coronaviruses. The low pathogenicity of alpha and beta coronaviridae (mainly responsible for common colds) did not make them a priority for vaccine research. When SARS-CoV-1 emerged in 2003, vaccines against this virus were tested in the preclinical phase and phase I in humans, but their industrial development was stopped with the spontaneous resolution of the epidemic (4). Vaccines against MERS-COV were tested for several years, but none reached the marketing stage (4, 5). While all this work did not result in vaccines used in humans, it allowed for the identification of the antigens of the coronaviruses targeted by our immune responses. Neutralizing human antibodies are directed against the Spike (S) protein (responsible for the particular crown aspect observed in structural studies of coronaviruses), and especially against one of its sequences called Receptor Binding Domain (RBD) (6). The S-protein is responsible for the invasion of human cells through interaction between its RBD region and, in the case of SARS-CoV-2, a specific receptor for the angiotensin 2 converting enzyme (ACE2) expressed by many human cell types, in particular in the pulmonary and vascular tissues. The S-protein was therefore selected as the main target against which an immunization by vaccine should be generated in order to obtain a protective immune response capable to hamper attachment and invasion by the virus the way natural antibodies do. Prior knowledge of these elements from related-coronaviruses studies largely contributed to accelerating the identification of SARS-CoV-2 vaccine targets and the determination of their corresponding genomic sequences (4, 7).

### Research on Immunological Responses Elicited by SARS-CoV-2 Infection in Humans and Other Primates

Although vaccine does not have to exactly reproduce the natural immunity, immunological studies conducted *in vivo* during infection by SARS-CoV-2 were also of great help to presume what should be ideally induced by vaccination.

Irrespective of the presence of symptoms, the virus induces production of specific antibodies, following a pattern similar to that observed in most viral infections: rapid production of IgM-antibodies (peak at 10 days) then rising of IgG with a peak around 20 days to decline onwards (8, 9). It is estimated that within 1 month of infection, over 90% of patients will have produced specific IgG (10). In asymptomatic patients—who initially produce fewer antibodies—specific IgG may be no longer detectable as early as 2 months after the infective contact (8), whereas, in some other people who generated a stronger immune response (often but not always associated with disease severity), the IgG could still be detectable up to 8 months later (11). How long would last the protection remains nevertheless unpredictable yet given the slight decline over time. Of note in the case of SARS-CoV-1, IgG antibodies were measured even more than 2 years after infection (12). The neutralizing antibodies are very specific and do not cross-protect against other coronaviruses. Besides the production of IgG antibodies, there is a production (then a decay) of IgA antibodies in the respiratory mucous membranes. These have been shown of major importance to prevent asymptomatic carriage and transmission of infection (13). Moreover, the Spike-protein stimulates the genesis of CD4 + lymphocytes, with a weaker effect on CD8+ lymphocytes. In addition to the Spike-protein, structural and non-structural regions of the nucleocapsid contribute to the stimulation of T cell responses and might be considered as additional targets for future vaccines especially to prevent escaping mutants (14). Unlike antibodies, there may be cross-reactivity on CD8+ lymphocytes between other epitopes from SARS-CoV-2 and from previously met coronaviruses, suggesting why some individuals could benefit from prior protective immunization (14). The development of a coordinated, specific adaptive immune response involving genesis of CD4+, CD8+, and neutralizing antibodies has been statistically associated with a milder pattern of infection while a suboptimal cellular immune response has been correlated with advanced age and worse outcome (15). In some individuals, however, the host immune responses can be amplified in such an uncontrolled manner that an inappropriate secretion of inflammatory cytokines will be triggered, which is responsible for major tissue damages (16).

In addition to human studies, experiments in other primates were of great use, especially at the beginning of the pandemic when the production of neutralizing antibodies against SARS-CoV-2 was demonstrated as well as their contribution to the resolution of infection in a macaque model (17). The observation that in primates a primary infection protects against reinfection (18) gave additional arguments to assume the efficacy of a vaccine, as did the evidence from laboratory assays of a human functional immune memory persisting months after infection (11, 19**)**. However, cases of re-infections (symptomatic and asymptomatic) have been reported for SARS-CoV-2 in humans (19) and also for MERS-CoV in animals (20), irrespective of the circulation of mutant strains. Only a few reinfection cases were well-documented on the immunological side by investigating the type and function of immune memory responses. In addition to the issue of escaping variants (21), the existence of reinfection questions the possibility of waning immunity as well as the role of memory cells and the way to efficiently induce them by vaccines. So far, there is no surrogate of protection allowing for identification of previously-exposed individuals at risk for re-infection, nor to quantify the duration of protection provided by the various vaccines. Comprehensive immunological studies allowing for the definition of standardized correlates of protection are importantly needed. Such studies will also be helpful to clarify concerns about the hypothesis of Antibody-dependent Enhancement of Disease (ADE) during which an aggravation of the disease linked to the production of facilitating antibodies induced after infection or by vaccination is observed (22). The ADE phenomenon has been well-documented for Flaviviridae like Dengue fever and mainly occurs when low antibody titers or low-affinity antibodies are produced. The reports of ADE in some animal models during trials of SARS-CoV-1 and MERS-CoV vaccines (23), as well as the observation that high antibodies rates correlated with the severity of outcome in COVD-19 patients (24) have raised concerns on safety and efficacy on futures anti-SARS-CoV-2 vaccines at the early stage of their development. Fortunately, this hypothesis is rendered unlikely for the moment considering the results of most clinical trials that did not demonstrate any case of ADE, neither after natural infection nor after vaccination of previously infected people. Nevertheless, until now, we do not benefit from any immunological assay or biomarker that is able to distinguish between a severe viral infection from an immune-enhanced disease (whatever this would be enhanced by antibodies, T cells, or innate-immunity pathways). Further in-depth investigation assessing the host immune responses and evaluating the risk of immunopathology after natural infection or vaccines will be of utmost importance to improve future prevention strategies, even now that vaccination campaigns have been globally rolled out.

### Prior Existing Vaccine Platforms and Regulatory Facilities Adapted to Emerging Virus

All the above information could not have been exploited in such an efficient way without the experience drawn from previous epidemics, which had already led to the creation of vaccine platforms, international collaborations and regulatory facilities (like emergency use authorization procedure) adapted to emerging viruses^1^. In common circumstances, the production and marketing of a new vaccine take more than 10 years. However, an epidemic setting requires shortening the duration of vaccine development stages by overlapping the phases by starting from the outset with a phase “1/2” followed by the launch of phase 3 if intermediate results appear favorable. Such a fastened procedure was implemented to develop the pioneering vaccine against the Ebola virus (25), for which a vaccination campaign could be started after 5 years only. Given the state of emergency triggered by the COVID-19 pandemic, the American [the Food and Drug Administration-(FDA)] and European [the European Medicines Agency-(EMA)] regulatory agencies and the WHO were immediately solicited to define the level of performance required to allow marketing of a SARS-CoV-2 vaccine: clinical efficacy of 50% (with a lower limit of confidence interval ≥ 30%) was set as a sine-qua-non condition for a vaccine to be considered beneficial to public health (26). For the most promising vaccine candidates, commercial production started well before the results of phase 3 were obtained. To support research, extraordinary funding has been granted by various governments and international associations allowing for the precious gain of time. The accelerator COVAX platform was built by the Global Vaccine Alliance (GAVI), the Coalition for Epidemic Preparedness Innovations (CEPI), and the WHO to promote research, development, and manufacture of many SARS-CoV-2 vaccine candidates at an affordable price; the aim is to offer equitable access to vaccination all over the world and thereby to provide a global solution to the pandemic (Fair Allocation Framework)^2^.

## Types of Vaccine and Current Results

It is common wisdom that having a safe and efficient vaccine remains the best way to control the COVID-19 pandemic. Among all the candidates in development (2, 3), some of them use traditional approaches like virus-inactivated or virus-live attenuated vaccines while others are based on more recent technologies like vectored-vaccines or mRNA vaccines, two innovations developed throughout this last decade. Table 1 displays the main platforms used for COVID-19 vaccine development with their respective specificities and inconveniences.

**Table 1:** Vaccine platforms used for COVID-19 vaccine.

**Vaccine platform**	**Subtype of candidate**	**Principles**	**Advantages**	**Inconveniences**
1) Modified virus-containing vaccines		• Well-known technology, used in many other vaccines • Injection of the virus itself after it has been rendered unharmful by various processes.		
	1.1 Weakened	• Attenuation of the replicative capacities of the virus by culture methods or genes deletion	1) Induction of a robust immune response against various viral antigens (not only the S protein) 2) Generate humoral and cellular specific immunity. 3) Intranasal formulations possible allowing for IgA formation and prevention of asymptomatic carriage	1) Containing weakened but live virus, posing risks of disease in immunocompromised individuals 2) Heavy manufacturing conditions due to use of live virus
	1.2 Inactivated	• Killing of the virus by heat.	1) No live virus avoiding the risk of disease	1) Need of an adjuvant to generate sufficient immune stimulation
			2) Induction of immune response against various viral antigens (not only the S protein)	2) Need for highly secured manufacture conditions due to manipulations on the virus
				3) Generate only humoral specific immunity.
2) Protein subunits vaccines		• Well-known technology, used for many other vaccines • Injection of viral surface proteins that have been prior recognized as immunogenic. Formulations differ by the parts of proteins used (i.e., the entire protein S or only its receptor-binding domain)	1) Very safe. No pathogen agent used so no risk of disease and a well-known procedure 2) Easier manufactures (recombinant proteins produced by bacteria, yeasts or cell culture	1) Need of an adjuvant to generate sufficient immune stimulation 2) Generate mostly humoral specific immunity.
3) Vectored vaccines		• Innovative technology applied for a decade to fight against other epidemic viruses (like Ebola) (19). • Sars-Cov2 gene(s) introduced in a different unharmful virus used as a vector to infect humans' cells. Host cells will produce the Sars-Cov2 antigens selected for immunization +/- new vector viruses.		
	3.1 Replicating vector	• The vector virus has been attenuated to lose its pathogenic capacity and modified to carry Sars-COV2 genes, but it remains able to replicate in infected cells. • Example of viruses used are Measles, VSV, New Castle virus…	1) Highly immunogenic 2) Generate humoral and cellular-specific immunity. 3) Intranasal formulations possible allowing for IgA formation and prevention of asymptomatic carriage	1) Containing weakened but live virus, so there is a risk of disease in immunocompromised individuals
	3.2 Non-replicating vector	• Deletion of some genes of the vector renders it unable to replicate in host cells. Most commonly used viruses are modified adenovirus (AdV5/AdV26, AAV) or animals' viruses (ChAdOx1…). Vectors are selected to minimize previous natural immunity. Some formulations contain also antigen-presenting cells.	1) Generate humoral and cellular-specific immunity. 2) Some schedule involving one single dose	1) Possible immunization against the vector virus leading to loss of efficacy (because of previous contact with related viruses or immunization between both doses). 2) No intranasal administration
4) Nucleic acid-based vaccines		• Innovative technology based on the delivery to human cells of the genetic information necessary to produce SARS-COV2 proteins selected as a target for immunization.	1) Generate humoral and cellular-specific immunity. 2) Easy manufacture (*in vitro*, without live viruses)	1) No intranasal administration
	4.1 DNA vaccine	• Selected viral genes are introduced into bacterial plasmids easy to reproduce in a sufficient amount. The vaccine contains plasmids that will enter thanks to a small electric shock (transfection) inside the human cell nucleus where they will be translate and lead to viral protein synthesis.	1) Very stable and easy to store	1) Necessity of material for electroporation 2) Less immunogenic than RNA vaccine
	4.2 mRNA vaccine	The genetic sequence corresponding to the viral protein is already translated into mRNA, which is immediately readable by the human ribosomes bypassing the nucleus steps. The mRNA is delivered inside human cells through lipid shells. This pioneer technology has been already studied for other viral vaccines (against ZIKA virus, HIV-1) in animal and human phase 1/2 trials and appears promising for therapy against metastatic cancers (27)	1) Highly immunogenic 2) No live virus, so no risk of disease even in immunocompromised people 3) No modification of the human genetic pool (no entry in the nucleus)	1) Very unstable product (storage at ≤ 20–70°C for a maximum of 5 days) 2) Limited data in humans (pioneer technology used for only a decade)^1^

In total, 15 vaccines are now evaluated in phase 3, whereas five have already achieved phase 4 (2). As of early December 2020, two vector vaccines and four inactivated vaccines were already approved by Chinese and Russian authorities and are now being distributed in these countries and partner ones. Out of these six candidates, only the Gamaleya National Research Centre published until now interim data of phase 3 clinical trial for its AdV5/AdV26 not-replicating-vectored-vaccine (Gam-COVID-Vac) (27). With the United Kingdom starting first, mass vaccination campaigns have been launched in many European countries, starting at the end of December 2020, using first mRNA vaccines (the Pfizer/BioNTech BNT162b2 mRNA vaccine and the Moderna mRNA-1273 vaccine) then also the Astra Zeneca/Oxford AZD1222 vectored vaccine—all three approved for use by the EMA ^3^. The candidate from Johnson and Johnson, which is part of the COVAX program, has now also been authorized for use in Europe, while the Gam-COVID-Vac, the Novavax, and the Curevac candidates are under EMA review. As detailed in Table 1, compared to the mRNA formulations, the vectored vaccines or protein recombinant vaccines require less stringent storage conditions (and a single dose schedule for the candidate of Johnson and Johnson), whereas the Gam-COVID-Vac applies a heterologous prime-boost strategy (see below).

Many publications assessing candidates at various stages are available but a comparison between performances of each vaccine is rendered complicated by the variability of design and methodologies applied. For example, in immunogenicity studies, the minimal inhibitory concentration used to estimate the capacity of antibody neutralization ranges from 50 to 100%. Since COVID-19 is a new disease, we do not yet benefit from validated immunological surrogates of protection (i.e., a threshold level of antibodies or neutralization functional testing) that will allow for standardized evaluation of vaccine effectiveness. The same problem arises when willing to compare clinical efficacy since most phase 3 studies only recorded symptomatic cases whose definitions are eminently variable.

At the time of writing this review, four clinical phase 3 trials have been published, enrolling each 20–40,000 healthy adult volunteers (plus 100 12–16-year-old adolescents in the Pfizer study). Pfizer/BioNTech study showed 95% efficacy (95% CI, 90.3–97.6), as assessed 7 days after the second dose (28) and recent data under review are reassuring about the protection conferred against two new variants (29). Along the same line, the trial from Moderna reported 94.1% efficacy (95% CI, 89.3–96.8%;) after two doses (30). The publication from the Astra Zeneca/Oxford team demonstrated a mean efficacy of 70% for its ChAdOx1-S not-replicating vectored-vaccine (efficacy of 90% for patients having been given half dose first then a full second after 1 month; the efficacy was 62% for those having received two full doses 1 month apart) (31). However, these results were obtained in people 18–55 years old so that restricted use was firstly recommended by National Immunization Technical Advisory Groups (NITAGs) of some countries. More data are thus warranted to evaluate efficacy in older individuals though this is expected by observation from the prior immunological study (32). Last published was the interim analysis of the phase 3 trial of the Gam-COVID-Vac that showed 91.1% efficacy (95% CI 83.8–95.1) against documented COVID-19 after two doses (27). The firm Johnson and Johnson has already announced its candidate provided 66% efficacy (72% in the US cohort) in preventing symptomatic, laboratory-confirmed COVID-19 from 28 days after injection with even higher efficacy against severe forms of infection and including against the south African variant from the B.1.351 Lineage (33).

Concerning safety data, all phase 3 trials enrolled thousands of participants, allowing for a good assessment of short-term adverse reactions, which are known to occur within 6 weeks after injection (34). No trial reported major adverse events. As for minor to moderate reactions, they appear more frequent in young people and after either the second dose for the mRNA vaccine or the first one for vectored vaccines. Rapidly, some concerns arose about allergic reactions following administration of the Pfizer vaccine, mainly due to the lipid envelop necessary to transport the nucleic acid. Despite the media impact, the rate of anaphylaxis observed so far was not estimated to be a major issue or cause of contraindication by the competent safety authorities and WHO, but caution is still advised (medical monitoring 15–30 min after injection) especially when administrating this vaccine to individuals with a history of a previous severe allergic reaction^4^ (35). Some warnings were also published about facial palsy after the Pfizer vaccine but a causal relationship could not be retained so far. As for the candidate from Astra Zeneca, concerns were raised after three cases of transverse myelitis occurred in the post-vaccine period, but any relationship with the vaccine administration was discarded for two of the three cases (31). However, for all candidates, the period of follow-up before approval was a fortiori very short (3 months maximum after the second dose and 6 months in total) due to the emergency state. If safety concerns seem low for the moment and far away from outweighing the benefits, awareness will be of major importance during the universal mass vaccination campaign. As for all previously licensed vaccines, enlarging the vaccinated population and the follow-up period will likely unmask the occurrence of very rare events (<1/10^5^-10^6^), as serious anaphylaxis reactions or neurological/auto-immune disorders. A much longer time is therefore needed to identify a true causal relationship in vaccine recipients. Implementation of an international surveillance system recording all secondary reactions is now of utmost importance to guide vaccination policies and has been launched by the WHO. The fundamental role of pharmacovigilance reporting systems has been recently emphasized by the warning raised by some European countries about serious blood clots events occurring in individuals shortly after reception of the AstraZeneca vaccine. Although rare, the incidence of this disorder has been found higher than expected in unvaccinated populations, in particular among young vaccinated women. At the time of writing this paper, the causality could not be formally established, but the problem is under thorough investigation by EMA experts and international surveillance is ongoing^5^. This concern should be all the more seriously considered that COVID-19 is associated with a high prevalence of thromboembolic complications for which an immunological origin through the formation of anti-platelets antibodies has already been hypothesized (36).

## Discussion and Key Questions

Vaccination has started in many countries, using various types of vaccines and schedules. However, important questions remain, and these should be addressed in the near future to ensure the success of the vaccination campaigns.

### What Could We Presently Expect in Terms of Effectiveness?

Until now, whatever was the studied candidate, the rate of efficacy published only reflected the individual rate of protection against disease (decrease in the number of patients getting symptomatic infections with a variable degree of severity, as compared to the placebo group). No data currently allow us to assess the impact on viral transmission, although expected according to mathematical modeling (37). Animal studies showed that neutralizing IgG reduces viral shedding in upper airways without however abrogating it (38). All phase 3 candidates induce circulating neutralizing IgG antibodies, but none of them have been proven to generate IgA antibodies that favor sterilization of the upper respiratory tract and therefore hinder the asymptomatic carriage of the virus (13). Such antibodies are preferentially generated when antigens are delivered intranasally, but only a few vaccines that are suited for intranasal administration have been developed, and even fewer have already entered in clinical trials.

Another key point is the duration of the induced protection, especially considering the lack of knowledge about anti-SARS-Cov-2 natural immune memory responses and the existence of reinfection with the same strain. The period of follow-up in the first published vaccine studies did not exceed 3 months after the second dose; hence we can wonder about the persistence of the induced immune responses (both cellular and humoral) in the mid- and long term and the need for additional booster doses. Whether the number of doses administered during the primary vaccination series could influence the robustness and duration of protection, remains another poorly documented issue.

On the same line, a discussion ensued about the maximum time interval between the two requested injections, originally designed to be 21 days for the Pfizer/BioNtech mRNA vaccine. This was based on the observation that specific immunity starts to be detectable 12 days after the first dose. Since numerous countries are facing a resurgence in the epidemic, notably due to the raising of more transmissible variants, the WHO and EMA have authorized to extend the interval between the two doses up to 42 days^6^. While delaying the second injection would not reduce overall efficacy after complete vaccination, the extended window period between the two doses could prolongate a suboptimal immunization status, insufficient to fully protect the recipients and perhaps favorable to the selection of escape mutants. It thus seems important to follow at best the originally recommended vaccination schedule and to postpone the second injection only if the circumstances absolutely require it. Individuals should be aware that they are not fully protected after a single dose and that control measures should absolutely not be relaxed. Creating an extended window period during which the immune response is suboptimal could furthermore constitute a theoretical risk factor for the development of ADE, which could mainly occur when low antibody titers or low-affinity antibodies are produced.

### Who Should Be Vaccinated?

Another major issue is to define the population to vaccinate. It is commonly admitted that vaccination should not be kept for all risk groups, this includes low, moderate and high risk groups [who obviously should be given priority (39)] but should be distributed to the highest number of people in order to slow down or even eradicate the circulation of the virus. Human history overflows with examples showing that controlling viral pandemic through vaccination is achievable, like for smallpox, poliomyelitis, and measles. However, it demonstrates also that as soon as the vaccine coverage becomes insufficient, outbreaks are observed (40, 41). The minimum rate of vaccine coverage requested to achieve suppression of community transmission and herd immunity is the function of each pathogen characteristic (way of transmission, incubation period, and fatality rate, which are all involved in the calculation of the basic and effective reproductive numbers) (42). This vaccination coverage rate was estimated around 60–70% for SARS-CoV-2, far from the >95% required to control measles. However, this estimation is susceptible to change over time since more transmissible variants are unfortunately emerging, the calculation also depends on social behavior and population heterogeneity, and as the first estimation implies that all infected (or vaccinated) individuals remain fully immunized for several months, which is uncertain especially regarding the possibility of asymptomatic carriage. Moreover, although host risk factors for severe COVID-19 are progressively identified (43, 44), it is basically impossible to predict who will develop serious forms of COVID-19 or its post-infective complications. Neither age nor the absence of comorbidity can guarantee a benign evolution of disease. The rise in incidence among children and young adults of a post-infectious multiple inflammatory syndrome (MIS-C) well illustrates this concern and sustains the universal mass vaccination policy (45). This way, fragile people who could either not quickly access the vaccine or who might not be eligible for vaccination because of their medical status will also benefit from protection thanks to the indirect effect and as will those ones who will only develop a suboptimal immune response.

At first glance, the solution seems straightforward: everyone without formal contraindication should be vaccinated to eradicate at most the human reservoir and hamper the circulation of the virus.

However, other questions arise. Firstly, should we somewhat adapt the schedule to subjects who have had a documented resolved infection or had been identified as a carrier? To date, no one can guarantee the duration and intensity of the protection conferred by the natural infection, although *in-vitro* indicators of immune memory have been found 6–8 months after infection (11, 46). Cases of re-infections (symptomatic and asymptomatic) have been clearly reported (19, 20), and the reinfection rate (defined as 2 positive PCR > 90 days apart with 7 days minimum without symptoms before the second sample) is currently estimated around 0.7–3.9% (47, 48). At the individual level, the decision to vaccinate could partly be guided by the serological status, pending more thorough testing assessing also cellular immunity will become available. If a large amount of antibody persists, the vacciney appears useless but follow-up testing could be advised. Vaccination could be indicated when the level of suspected neutralizing antibodies declines significantly. However, no cut-off has been validated, and assessment of the immune status of all vaccine recipients constitutes an unrealistic scenario implying carrying out serological testing on a large scale and spending considerable logistical and financial resources. Since the ADE hypothesis is not supported to date by clinical trials results (including previously infected people), and since series of data seem to indicate that most individuals are protected at least until 3–6 months after a documented infection (49), providing the vaccine after this delay appears a wise option. The vaccine is then expected to act as a booster, helping to mount a faster immune response in case of further contact and reinforcing immune memory. As supported by some recent immunological studies (50, 51), a single dose schedule might be sufficient in previously infected people and is now proposed by some regulatory agencies^7^. Of note, according to our opinion, the benefits from vaccination remain a matter of debate in subjects who presented with a severe form of COVID-19 with cytokines storm, for which the greatest precautions should probably be taken before reintroducing any SARS-CoV-2 antigen. Individuals with a history of severe COVID-19 were actually excluded from phase 3 trials, and much more data are needed to guide this decision. As well, knowledge of the serological status could be helpful in particular subgroups of more fragile individuals such as the elderly, more prone to develop ADE, to tailor the number of doses in case of prior infection. Again, data from phase 3 trials concerning extreme age groups are still awaited. These groups obviously deserve specific attention considering particular features of their immune systems, like the well-documented immunosenescence phenomenon characterized by lower immune responses to several vaccines in the elderly. Moreover, it has been found that anti-SARS-CoV-2 T cell responses are disrupted after the age of 65 years (15). Since the elderly are at the highest risk for life-threatening COVID-19, almost all countries have decided to launch their vaccination campaign by giving them absolute priority, especially for those living in care homes. Further assessment of efficacy and safety is still ongoing in this cohort and will be important in order to tailor the vaccine schedule if necessary (interval and number of doses or amount of antigens). Moreover, a deeper investigation into the scarcity of cellular immune responses observed in elderly people exposed to SARS-CoV-2 could have important implications to guide the design of future new vaccines against this virus and other related ones.

What about the other extreme age group: children? Unlike other respiratory viruses, children are less susceptible to COVID-19 than adults are. Not only do they present with milder forms of infections (52), but they seem less likely to become infected after exposure (especially for the youngest) (53, 54). Adolescents, however, show the same features of transmission and disease as adults. Many studies are ongoing to assess to which extent children contribute to the spread of the SARS-CoV-2 pandemic and the reasons why they are less susceptible. If it is generally admitted that the children (especially until primary school age) are not the motor of transmission, they can still transmit the disease once infected, irrespective of their age (52, 55) and this is all the more difficult to estimate that they are often asymptomatic carriers. A reflection should therefore be carried out on whether, once vaccination of priority groups is be completed, children should also be considered for vaccination and if so, for which age group. Regarding features of infection and transmission, consideration should probably be given to adolescents first and then to school-age children as well as those with comorbidities irrespective of their age. The main goal would be to decrease the circulation and reservoir of the virus inside the community, especially if willing to achieve an optimal vaccine coverage, provide herd immunity and prevent the rapid spread of more transmissible new variants, which showed increased infectivity also among children (56). Although it should be stressed out that children represent only 17.4% percent of the EU population and <2% of hospitalized COVID-19 cases, they constitute a very dynamic part of the population, even beyond their school and household, by traveling and gathering during collective activities and have regular contacts with their grandparents. Compliance with social distancing measures is also more difficult to achieve in young individuals. Some popular waves are pushing now to vaccinate in priority young adults and adolescents, whom the psychosocial burden of the pandemic is estimated to be among the highest after health care workers and elderly (57). The increased incidence of MIS-C in the pediatric population this summer as well as the existence of severe cases (though rare) within the youngest is an additional argument to consider for vaccination in the mid- or long term if high epidemic circulation is still ongoing. Moreover, co-infections with SARS-CoV-2 and other respiratory viruses like Influenza or RSV have been described to lead to severe pneumonia (58). Even though winter epidemic viruses were almost absent from the landscape this year, we can hypothesize that a problem could arise once others respiratory viruses will come back and affect the youngest population again. RSV and influenza are major causes of morbidity and hospitalization every year in pediatrics, and no one can predict what could give co-infection with SARS-CoV-2, especially for infants and children with comorbidities.

Besides the encouraging results of the mRNA Pfizer vaccine in hundreds of adolescents, data on vaccine efficacy and safety are awaited in children who usually presented with higher immune responses. If considering vaccination in pediatric groups in the future, the number of doses and the optimal amount of antigen should be determined for each age category in order to maximize efficacy but also to minimize the risk of reactions (like fever, pain, rash, etc.). It should also be determined to which extent the history of atopia (a frequent problem in pediatrics) requires more caution or constitute a contraindication. Last but not least, a place should be found in the already tight vaccine schedule, without hampering compliance to other vaccinations and in respecting intervals with other injections to minimize adverse events.

Choosing the vaccine candidates that are the most adapted for children might be a crucial point in this debate and could differ from these for adults. The ideal vaccine for pediatric setting should, besides offering optimal protection and long-term immune memory, be not too immunogenic, be administered following a single dose schedule, be suitable for intranasal delivery (no needle and prevention of carriage frequent in the youngest), require no strict storage conditions, and, if possible, provide simultaneous protection against other viruses whose others vaccines could then be avoided.

Finally, the question of pregnant women and immunocompromised patients deserves specific attention. Whereas, formulations containing live replicating viruses have formally to be avoided, no data are available for mRNA vaccines in these cohorts. As for not replicating vectored vaccines, the precaution principle should prevail while waiting for further recommendations. Risk assessments of COVID-19 in pregnant women have given conflicting results considering the rate of serious infections, hospitalization, and complications like preterm delivery (59, 60). Pregnant women are however considered as a risk group by the CDC^8^. Even if no specific physio-pathological argument or animal study raises concerns regarding mRNA vaccination in this cohort, the WHO and the EMA do not recommend systematic vaccination given the absence of specific data but rather a case-by-case approach with cost-benefit assessment, especially for women belonging to other risk groups^1^^,^^6^^,^^9^^,^^10^. It should be highlighted that vaccine studies including pregnant women are definitely needed if willing to provide reliable recommendations in the near future. For women who are breastfeeding, a recent EMA report indicated that no particular risk should be considered for the mRNA vaccine, due to quick degradation of the product that is not suspected to be armful once entering the digestive tract of the newborns. For persons living with HIV or other immunocompromising comorbidities, as long as they are treated and stable, and given they are at higher risk of severe COVID-19, vaccination is recommended after medical advice^1^^,^^6^^,^^9^. However, not all types of vaccine would be acceptable in this cohort since no live virus could be administered. Protein subunit or mRNA vaccines would therefore be preferred.

### How Do We Choose Between the Different Vaccines?

Table 1 displays the different types of vaccines, each offering various advantages and inconveniences. Until now, their use depends on the performances achieved as well as on marketing authorization earned from regulatory agencies and commercial agreements. Some formulations may better suit some settings than others depending on their conditions of their supply, storage, and schedule of administration. However, equity and accessibility for all must be protected, and research is encouraged to provide the best candidate vaccine for each socioeconomic and geographical situation.

All vaccines are directed at least against epitopes of the S protein or its RBD sequence that either vectored or presented in different ways, could be theoretically used in a “heterologous prime boost strategy.” This strategy consists of giving two doses of vaccine where each belongs to a different formula and therefore presenting the antigens differently. This process seems to induce a higher immune response than using the same formulation twice (61). The heterologous prime boost has already shown promising results in vaccination against HCV and HBV. Such a strategy could be of great interest against SARS-CoV-2, but further studies are needed to investigate its superiority and harmlessness in humans and animals.

Finally, the choice of vaccine type should be continuously evaluated in the future to fit at most the host and the pathogen. If necessary, adaptations should be envisaged for subgroups of individuals according to their age, immune and medical status, and history of allergy or pregnancy. Private–public partnerships would facilitate the establishment of broad international cohorts, which is mandatory to monitor vaccine effectiveness and safety among individuals suffering from rare conditions.

### Adaptation of Vaccine to Viral Evolution

Last but not least, the success of universal mass vaccination also relies on the implementation of continuous surveillance of circulating viral strains as well as of an active reporting system of cases to identify vaccine failure. Like all RNA viruses, the SARS-CoV-2 genome undergoes frequent spontaneous mutations or deletions that are fortunately less frequent than other RNA viruses due to the presence of a corrective enzyme (62).

Whereas, not every mutation leads to consequences on pathogenicity, some could be the source of trouble, either through increasing virulence or transmissibility or by impairing the protection achieved by prior infection or vaccination (62). Such events could happen when significant mutations occur in genes encoding the S-protein: the main target of the majority of vaccines. As seen with many other pathogens, new variants can outcompete the local dominant clone (s) because the acquired mutations confer selective advantages for survival and dissemination.

From the beginning of the pandemic, numerous SARS-CoV-2 variants characterized by mutations on surface proteins compared to the original strain isolated in Wuhan in December 2019 have been identified within the forefront the D614G mutated strain that early became dominant in Europe and the Americas (63). Further new variants have recently been identified spreading all over the world (62, 64). At the time of writing this paper, the most harmful variants in Europe either belong to the lineage B.1.1.7 (UK variant VOC 202012/01) or to the lineage B.1.351 that originated from South Africa (48, 61). Both of them harbor mutations affecting the sequence of the S-protein, of which one (N501Y) affects its RBD. These mutations are hypothesized to increase viral affinity for human cell receptors and facilitate replication, leading to higher transmissibility (64). Fast recrudescence of cases has actually been observed with these strains (47, 56), requiring the implementation of more stringent lockdown measures in some regions. Though data are conflicting, results from Britain epidemiological reports tend to indicate increased severity of infection with the B.1.1.7 mutant (48, 56). Fortunately, according to preliminary immunological studies, the genetic changes found in this variant seem only to marginally affect the efficacy conferred by currently available vaccines (29, 65). However, real concerns exist about the protection against the South-African and Brazilian variants that both carrying the mutation E484K believed to impair the neutralizing capacity of vaccine-induced antibodies (64, 65). Strikingly, this mutation has been identified additionally in some B.1.1.7 UK strains that will now deserve particular attention and monitoring of cases. The Brazilian variant (P.1 lineage) has been first reported in the city of Manaus (as well as in some travelers in Japan and South Korea), creating an important upsurge of cases in this city thought to have, however, reached a high level of community immunization. Only a minority of cases have been reported to date in Europe and are mostly associated with travel history, but further monitoring is required.

Since mutations belong to the natural dynamic evolution of RNA viruses, it seems likely that several other SARS-CoV-2 variants will emerge over time, with more or fewer implications on pathogenicity and transmissibility but requiring constant assessment of vaccines effectiveness and perhaps adaptation of the presented antigens to enlarge protection. A similar model is -already applied with the Flu vaccine in which vaccination must be repeated yearly and vaccine production adapted anticipatively according to the most likely antigenic drifts for the four dominant influenza A/B strains. International collaboration and elaboration of a reference database are crucial to identify new lineages and understand the implications of mutations on pathogenesis and on protection confer by the available vaccines. Whereas, effects of new mutations on disease severity remain uncertain to date, we can wonder whether future genetic variations in SARS-CoV-2 associated with host immune adaptations will result in persisting seasonal epidemics with, however, a less serious pattern of infection, like observed for H1N1 for almost a century (66).

## Conclusion and Perspectives

In nearly 15 months, SARS-CoV-2 has been responsible for a dramatic burden of disease and a global economic recession. To date, the collective immunity achieved is largely insufficient, as evidenced by the persistence of the pandemic, and the physical distancing and hygiene measures, while mandatory to avoid overflow of the healthcare system, are not enough on their own to control the spread of the disease especially in a long-term perspective. The emergency state generated by COVID-19 sparked important rallying all over the world, which, in addition to the experience drawn from prior viral epidemics, allowed for faster development of a vaccine.

Broadly vaccinating the population remains the best way to fight COVID-19 even if additional data are needed to better tailor vaccine schedules (notably for particular subgroups and previously ill people) and identify long-term side effects. Many promising options, like new vaccine candidates and prime boost strategies, are still under investigation.

Continuous monitoring of the circulating viral strains, associated with the international post-vaccination surveillance system reporting host infections and reactions, will be the cornerstones to ensure effectiveness and safety for everyone. A judicious choice of the best formulation, based on economic and logistical constraints but also on scientific and medical arguments, could help to optimize the success of vaccination campaigns worldwide in addition to constant evaluation of vaccine effectiveness on new variants to avoid breakthrough infections.

Control of the pandemic will, however, only be achieved through international coordination on preventive strategies and vaccination policies and if social distancing and hygiene measures are kept long enough while reaching sufficient vaccine coverage to interrupt viral epidemic circulation.

## Author Contributions

SB and PD contributed equally to this article in reviewing the literature and writing the manuscript. All authors approved the submitted version.

## Conflict of Interest

The authors declare that the research was conducted in the absence of any commercial or financial relationships that could be construed as a potential conflict of interest.

## Footnotes

^1^https://www.fda.gov/vaccines-blood-biologics/vaccines/emergency-use-authorization-vaccines-explained

^2^https://www.who.int/initiatives/act-accelerator/covax

^3^https://www.ema.europa.eu/en/human-regulatory/overview/public-health-threats/coronavirus-disease-covid-19/treatments-vaccines/covid-19-vaccines

^4^https://www.gov.uk/government/publications/regulatory-approval-of-pfizer-biontech-vaccine-for-covid-19/information-for-healthcare-professionals-on-pfizerbiontech-covid-19-vaccine

^5^https://www.ema.europa.eu/en/news/covid-19-vaccine-astrazeneca-benefits-still-outweigh-risks-despite-possible-link-rare-blood-clots

^6^https://www.who.int/news-room/events/detail/2021/01/05/default-calendar/extraordinary-meeting-of-the-strategic-advisory-group-of-experts-on-immunization-(sage)-−5-january-2021

^7^https://www.has-sante.fr/jcms/p_3237271/fr/strategie-de-vaccination-contre-le-sars-cov-2-vaccination-des-personnes-ayant-un-antecedent-de-covid-19

^8^https://www.cdc.gov/coronavirus/2019-ncov/need-extra-precautions/pregnancy-breastfeeding.html

^9^https://extranet.who.int/pqweb/vaccines/who-recommendation-covid-19-mrna-vaccine-nucleoside-modified-comirnaty%C2%AE

^10^Available online at: https://www.ecdc.europa.eu/en/covid-19-pandemic
